# Targeting Mycotoxin Toxicity: From Molecular Mechanisms to Nutritional Interventions

**DOI:** 10.3390/vetsci13050421

**Published:** 2026-04-26

**Authors:** Shirui Huang, Yiqin Gao, Thobela Louis Tyasi, Abdelkareem A. Ahmed, In Ho Kim, Hao-Yu Liu, Saber Y. Adam, Demin Cai

**Affiliations:** 1 Laboratory of Animal Physiology and Molecular Nutrition, Jiangsu Key Laboratory of Animal Genetic Breeding and Molecular Design, College of Animal Science and Technology, Yangzhou University, Yangzhou 225009, China; 241902509@stu.yzu.edu.cn (S.H.); 241902506@stu.yzu.edu.cn (Y.G.); 007725@yzu.edu.cn (H.-Y.L.); 2 Department of Agricultural Economics and Animal Production, School of Agricultural and Environmental Sciences, University of Limpopo, Private Bag X1106, Polokwane 0727, South Africa; louis.tyasi@ul.ac.za; 3 Biomedical Research Institute, Darfur University College, Nyala 63313, South Darfur State, Sudan; kareemo151@gmail.com; 4 Department of Veterinary Sciences, Faculty of Animal and Veterinary Sciences, Botswana University of Agriculture and Natural Resources, Gaborone P.O. Box 100, Botswana; 5 Department of Animal Resource and Science, Dankook University, Cheonan 31116, Choongnam, Republic of Korea; inhokim@dankook.ac.kr

**Keywords:** mycotoxin, feed contamination, feed security, animal health

## Abstract

Mycotoxin contamination is an important threat to food and feed safety as well as human and animal health, with particular emphasis on oxidative stress, apoptosis, autophagy, inflammation, and dysbiosis. Mycotoxins represent major health threats because they disturb cellular homeostasis and induce oxidative damage. Nutritional factors, such as dietary antioxidants and bioactive chemicals, can influence the body’s reaction to mycotoxin exposure, either reducing or increasing its effects. This study discusses how mycotoxins (aflatoxin B1, deoxynivalenol, and ochratoxin A) induce oxidative stress by producing reactive oxygen species (ROS)-mediated DNA damage, which induces cellular damage and activates apoptosis, an intended cell death process that is critical for tissue integrity. Furthermore, mycotoxins alter autophagy, a cellular degradation process that can be beneficial or destructive depending on the situation, affecting cell survival. The inflammatory response is particularly important because mycotoxin-induced oxidative stress and cell damage activate inflammatory pathways, which contribute to tissue injury and disease progression. Nutritional factors high in antioxidants, anti-inflammatory substances (Lycopene, Curcumin, Thyme oil, Gum Arabic, and Ginger), probiotics, and prebiotics show potential in mitigating these negative consequences by reducing oxidative stress and inflammation. Advances in molecular biology and omics technologies (transcriptomics, proteomics, metabolomics, and single-cell sequencing) can lead to better knowledge of the underlying pathways, allowing for more tailored nutritional recommendations and medicinal interventions. Finally, combining dietary modulation with mycotoxin risk management is a viable path for protecting health and increasing resilience to mycotoxin-related toxicities in animals.

## Simple Summary

This review highlights the serious threat that mycotoxin contamination poses to food and feed safety and health, particularly by producing oxidative stress, apoptosis, autophagy, and inflammation and dysbiosis. Mycotoxins (aflatoxins, deoxynivalenol, and zearalenones) disrupt cellular homeostasis and induce oxidative damage through the generation of reactive oxygen species (ROS), leading to cellular injury and death. Mycotoxins also affect autophagy, which influences cell survival, and they stimulate inflammatory pathways, causing tissue damage. Nutritional variables, particularly antioxidants and anti-inflammatory substances (Lycopene, Thyme oil, and Gum Arabic), can help to reduce these consequences by lowering oxidative stress and controlling cell death and inflammation. This review highlights the potential of dietary methods for protecting against mycotoxin-induced toxicity and suggests additional research into beneficial dietary components using molecular biology and omics technology (transcriptomics, proteomics, and single-cell sequencing). Combining dietary therapy with mycotoxin risk management is a potential strategy for improving health resilience and mitigating toxicity consequences.

## 1. Introduction

Mycotoxins are toxic secondary metabolites produced by fungi that are widely found in the natural world. Mycotoxins represent a major risk to global food safety and security [1,2,3]. There are currently around 400 known mycotoxins, although only a small number of them are regulated worldwide [4]. The most significant mycotoxin categories are aflatoxins (AFBs), trichothecenes (T-2s), zearalenones (ZENs), deoxynivalenol (DON), ochratoxins (OTAs), ergot alkaloids, fumonisins, and patulin. These are generated primarily by *Aspergillus*, *Claviceps*, *Fusarium*, and *Penicillium* species [5]. Numerous agricultural products, including dairy products, dried and fresh fruits, spices, traditional Chinese medicinal herbs, and staple crops like corn, wheat, barley, and peanuts, contain mycotoxins. In addition, samples frequently evaluated by the Alltech 37+ Analytical Laboratory (Nicholasville, KY, USA) demonstrate not only the presence of various mycotoxins but also a high prevalence of emerging mycotoxins (Table 1) [6]. Emerging mycotoxins are not classified within the traditional categories of mycotoxins, such as AFB or DON, and are identified neither as those that are not frequently assessed nor subject to legislative regulation [7]. Mycotoxin contamination can happen at any point in the supply chain, including field cultivation, harvesting, storage, processing, and shipping. When poor storage conditions are used, the danger of contamination increases considerably [8,9,10]. In fact, infection by a single mycotoxin is comparatively uncommon; instead, many mycotoxins frequently coexist. According to national studies, feeds frequently contain AFB1, DON, and ZEN, either alone or in combination [11]. Such combined contamination may have additive or synergistic effects, which could have more severe negative consequences on the production and health of animals [12]. Beyond these combined toxic effects on animal production and health, mycotoxin exposure also leads to severe organ-specific damage and disease.

Mycotoxin exposure can be detrimental in a number of ways [13]. In addition to their potent toxicity, mycotoxins can induce cancer, teratogenesis, and mutagenesis. According to research, the liver, kidneys, oral cavity, gastrointestinal tract, spleen, brain, and nervous system are among the organs that are most targeted by mycotoxins [13,14]. Since the liver is the site of mycotoxin detoxification, AFB_1_ often primarily impacts the liver. T-2 mostly damages the gastrointestinal tract and oral cavity [15], whereas OTA primarily affects the kidneys. Livestock and poultry that eat feed contaminated with mycotoxins show a number of disease indications. Pigs, for instance, may have negative symptoms like vomiting, diarrhea, weakened immunity, and stunted growth and development following consumption of contaminated feed. The intestine, the primary target organ of mycotoxins, is seriously damaged, which impairs intestinal barrier function as well as nutritional absorption and digestion. Different mycotoxins have different toxicity characteristics [16,17]. The most prevalent mycotoxins in feed and its raw materials are AFB_1_, DON, and ZEN, which have high carcinogenicity, immunotoxicity, and reproductive toxicity, respectively [11]. Beyond the direct toxic effects on animal organs and health, mycotoxin contamination also imposes a heavy economic burden on the agricultural and livestock industries.

Mycotoxin contamination not only affects animal health but also results in significant financial losses. This mostly results in direct agricultural product losses, diminished animal industry output, and obstructed international trade [18,19]. The Food and Agriculture Organization of the United Nations (FAO) reports that every year, mycotoxin contamination affects about 25% of the world’s crops to varied degrees, causing the losses of hundreds billions of US dollars [14]. Two modeling methodologies, one utilizing operating characteristic (OC) curves for sampling and testing, and the other applying partial equilibrium economic analysis, estimate that AFB contamination may result in losses to the US corn industry between USD 52.1 million and USD 1.68 billion annually, especially if climate change leads to increased contamination in the Corn Belt, as observed in warmer years such as 2012 [20]. The enormous range reflects the natural year-to-year variability in contamination levels, with higher losses representative of warmer years. Incomplete data from China’s National Food and Strategic Reserves Administration indicate that mycotoxin contamination induces 6.2% of the country’s annual grain loss, which is more than six times the amount of grain increase needed to guarantee national food security. The livestock industry suffers significant financial losses as a result of the loss of feed nutritional components brought on by mycotoxin contamination, which is particularly detrimental to animal health and productivity [21]. Excessive mycotoxin levels are now a major barrier to the export of agricultural products in international trade. More than 100 countries or regions have developed mycotoxin limit standards and regulations as a result of the ongoing improvement of international food safety standards. The limit standard values are continuously declining, which raises the requirements for the import and export of agricultural products. It is important to remember that mycotoxin contamination results in significant indirect losses as well as direct financial losses, including emergency rescue, medical costs, post-event compensation, and the mortality of livestock and poultry.

Mycotoxins are difficult to eradicate once they have been generated; therefore, current control measures generally focus on preventing toxin generation and reducing human and animal exposure to toxins. Nutritional interventions are largely mitigative rather than curative, and at present, there are no broadly effective and economically feasible nutritional strategies that can fully counteract mycotoxin toxicity. The objective of this review is to identify and evaluate specific dietary components that can protect against mycotoxin-induced oxidative, inflammation and dysbiosis, using molecular biology and omics technologies to elucidate underlying protective mechanisms and inform tailored nutritional strategies for health risk reduction. Future studies might employ multi-omics techniques to identify predictive biomarkers for early diagnosis and intervention efficacy; to conduct translational research that links animal models to human populations, particularly in chronic low-dose exposure scenarios; and to investigate green detoxification solutions, such as designed enzymes and microbial breakdown, with no influence on nutritional quality. Addressing these gaps will move the research from mechanistic understanding to evidence-based nutritional treatments for reducing mycotoxin toxicity.

## 2. Mycotoxin Poisoning in Livestock, Poultry, and Pets

In a survey in northern Spain, 19 mycotoxins were biomonitored in the plasma of food-producing animals, such as cattle, poultry, pigs, and sheep, demonstrating extensive exposure [22,23]. Another study in Spain discovered mycotoxins, including aflatoxins B1, B2, G1, and G2, ochratoxins A and B, ZEA, DON, and sterigmatocystin, co-occurring in compound feed for cattle, pigs, poultry, and sheep [23]. The toxicity mechanisms of these substances are complicated and frequently involve molecular interactions that alter cellular activities, resulting in reduced animal performance, increased susceptibility to diseases, and large economic losses in livestock production [24,25].

One of the most significant impacts of mycotoxins is hepatotoxicity, which is especially common with AFB. AFB_1_, for example, is a highly genotoxic hepatocarcinogen. It can affect cytochrome P450 metabolism and lead to the creation of DNA adducts, causing liver damage and increasing the risk of cancer in mammals [26]. This is a serious problem since mycotoxin accumulation can impair liver detoxification mechanisms. Another significant adverse effect is immune suppression, which is aided by DON. DON reduces protein synthesis through ribotoxic stress, which can impair immunological responses, reduce vaccine efficiency, and increase an animal’s susceptibility to various infectious diseases [27]. Mycotoxins can impact both innate and adaptive immune functions, compromising the animal’s ability to fight off pathogens [28]. Reproductive toxicity is usually associated with ZEN, which mimics estrogen and induces hyperestrogenism, infertility, and other reproductive abnormalities in animals. ZEN may cause serious damage to the reproductive system, gastrointestinal system, and immunological organs. The global pollution status and risks posed by ZEN are well documented, as are numerous degrading strategies and dietary techniques used to minimize their impacts [29]. Fumonisins block sphingolipid production, which can cause neurological diseases such as horse leukoencephalomalacia. Other mycotoxins, such as OTA and beauvericin (BEA), have been reported to induce cytotoxicity in neuroblastoma cell lines, emphasizing their potential impact on neurological health [30]. Mycotoxins such as Fumonisin B1 (FB1) and OTA can cause ROS generation, resulting in cell cycle abnormalities and enzymatic activity changes in human neuroblastoma cells, indicating neurotoxic potential [31].

Beyond these specific organ toxicities, mycotoxins can alter nutrient digestion, absorption, and metabolism, resulting in reduced feed efficiency, decreased growth rates, and overall impaired animal performance [24]. These subclinical impacts are generally overlooked, although they contribute significantly to economic losses in the livestock industry. The intensity of mycotoxin effects can be impacted by metabolic changes between species and ages, such as glucuronidation and epoxidation. For example, chickens are particularly susceptible to mycotoxins, which can manifest as oxidative stress, compromising development, immunity, and overall health [32,33,34]. Mycotoxicosis in poultry often appears as poor feed conversion, mouth lesions (particularly from AFB), decreased hatchability, and immunosuppression, making them more sensitive to secondary infections [33]. Companion animals, such as dogs and cats, are increasingly considered family members, and thus the safety, appropriateness, and efficacy of their feed is critical. Pets are also susceptible to mycotoxins through contaminated dry feed, as grains and plant-based feedstuffs are common ingredients [35]. The presence of mycotoxins in pet feed poses major health concerns to pets and creates emotional and financial distress for owners [36,37]. However, mycotoxins are a common biological hazard in livestock production, with far-reaching consequences for health and productivity (Table 2). These negative consequences highlight the crucial need for effective prevention, detection, and mitigation techniques to protect animal health and maintain feed safety.

## 3. Cellular and Molecular Toxicity Mechanisms of Mycotoxins

### 3.1. Oxidative Stress Mechanism

Oxidative stress is the process in which the generation of intracellular ROS exceeds the scavenging capacity of the endogenous antioxidant system, resulting in an imbalance between oxidation and reduction states, attacking biological macromolecules and disrupting cellular homeostasis [66,67]. Extensive toxicological research reveals that oxidative stress induction is a common and important molecular mechanism behind the cellular damage and systemic toxicity of various mycotoxins, despite their differing chemical structures and target organs [14]. This section elaborates on the toxic mechanisms of mycotoxins from three perspectives: the induction of oxidative stress, downstream cellular events, and critical molecular pathways.

Mycotoxins induce intracellular oxidative stress via two primary methods, direct or indirect, indicating the origin of their toxicity. Various mycotoxins can directly interfere with the proper operation of mitochondria, the cellular energy metabolism center, resulting in excessive formation of ROS [68,69]. For example, DON can induce mitochondrial oxidative stress by activating the MAPK7/AhR/STAT3 signaling axis [70]. Treatment of mouse neuroblastoma cells (N2a) with T-2 generates a large increase in intracellular ROS levels and aberrant mitochondrial membrane potential, which is a crucial event initiating subsequent apoptotic pathways [71]. According to research, mycotoxins, in addition to increasing ROS generation, damage the cell’s ability to defend itself. Their principal targets include essential intracellular antioxidants and antioxidant enzymes, such as inducing glutathione (GSH) depletion [72]. Furthermore, mycotoxins inhibit the activity of antioxidant enzymes such as superoxide dismutase (SOD) and catalase (CAT) [73]; SOD, CAT, and glutathione peroxidase (GSH-Px) are the primary components of the antioxidant enzyme system [74]. Combined mycotoxin exposure can induce a simultaneous reduction in the enzymatic activity and gene expression levels of SOD and CAT in the livers and kidneys of mice [75]. T-2 coupled with AFB1 can drastically affect the activity and expression of enzymes like GSH-Px in cells [76]. Mycotoxins quickly induce oxidative stress in cells by “increasing supply” (raising ROS) and “restricting clearance” (reducing detoxification capacity), opening the door for further cascade damage.

Long-term oxidative stress induces a number of irreversible cellular impairments that eventually result in cellular malfunction or death. Polyunsaturated fatty acids in cell and organelle membranes can be attacked by ROS, particularly hydroxyl radicals, which can trigger lipid peroxidation chain reactions and produce end products such as malondialdehyde (MDA) [77]. Research demonstrates that MDA levels in mice’s serum, liver, and kidneys are markedly increased following gavage with mixed mycotoxins [78]. MDA content is also markedly increased when cells are treated with both AFB1 and T-2 [76]. Membrane fluidity, integrity, and selective permeability are directly disrupted by lipid peroxidation, which triggers cellular contents to seep out and organelles to malfunction. Additionally, ROS can directly target DNA molecules, resulting in base alterations and single-strand or double-strand breaks. This type of oxidative DNA damage is a key mechanism underlying the carcinogenic potential of mycotoxins such as AFB1 [79]. Furthermore, almost all intracellular physiological processes can be impacted by ROS-induced protein oxidation. In the end, programmed cell death is brought on by extreme oxidative stress. The mitochondrial process is very important: when ROS damage mitochondria, the membrane potential collapses, cytochrome C is released, and Caspase-9 and Caspase-3 are activated to carry out apoptosis. Research on T-2 toxin-induced apoptosis in N2a cells clearly demonstrates this pathway: the Bax/Bcl-2 ratio rises and Caspase-3/9 is activated due to the elevated ROS levels and decreased mitochondrial membrane potential [71]. Interestingly, that study also discovered that cellular autophagy was activated, and improved autophagy helped clear damaged components to some extent, relieving oxidative stress and apoptosis, showing the complex feedback processes that cells use in response to toxin damage.

Oxidative stress is not a single event; it multiplies or executes its adverse effects by modulating several cell signaling pathways. The Nrf2/Keap1 pathway is the most important defensive mechanism against oxidative damage [80,81,82]. Under oxidative stress pressure, the transcription factor Nrf2 is activated and translocates into the nucleus, commencing the transcription of a series of Phase II detoxifying enzymes and antioxidant proteins, which can be considered the cell’s compensating protective reaction [76]. However, if oxidative damage is severe enough, this protective system may fail. Simultaneously, ROS is a powerful stimulator of signaling pathways such as the MAPK family [71], which can further link oxidative stress signals to apoptosis execution mechanisms. Multiple mycotoxins are frequently found in practical feed and food contamination, and their combined toxicity often has synergistic consequences. Research reveals that AFB1 and T-2 toxins can induce synergistic toxic effects on cells, causing more severe decreases in cell viability and oxidative damage when combined compared to individual toxins [71]. This implies that composite exposure effects must be fully considered when determining mycotoxin threats.

Mycotoxins induce oxidative stress by directly stimulating ROS generation while also impairing the antioxidant defense system. This process damages biological macromolecules such as lipids, proteins, and DNA, interfering with important signaling pathways such as Nrf2 and MAPK, resulting in cellular malfunction, apoptosis, or necrosis. This central process provides a unifying framework for comprehending the broad toxicity of mycotoxins and suggests possible targets for developing antioxidant-based detoxification methods. Future studies should concentrate on the effects of low-dose, long-term composite exposure to more accurately assess their health risks.

### 3.2. Apoptosis and Autophagy Mechanisms

Apoptosis and autophagy, two highly regulated cellular processes, have been identified as key mechanisms underpinning the cellular and molecular toxicity of mycotoxins in recent years because of developments in cell death biology. These two processes are closely linked to oxidative stress and do not exist independently. Together, they comprise a complex regulatory network that controls the fate of cells.

Mycotoxins are strong inducers of apoptosis, especially trichothecenes like the T-2 toxin and DON and AFB1. The mitochondrial pathway, also known as the intrinsic pathway, is one of the apoptotic pathways’ primary channels of activity [83,84]. Mycotoxin-induced oxidative stress is frequently the first step in this process. For instance, administering T-2 to mouse neuroblastoma cells (N2a cells) first induces a notable rise in intracellular ROS [71]. In both N2a cells and bovine intestinal epithelial cells, excessive ROS damage mitochondria, resulting in a reduction or collapse of the mitochondrial membrane potential, an early important indicator of malfunction. When membrane potential is lost, the mitochondrial permeability transition pore opens, allowing pro-apoptotic substances such cytochrome C to enter the cytoplasm. The “apoptosome,” which is formed by cytochrome C, Apaf-1, ATP/dATP, and pro-caspase-9, activates the initiator caspase-9. Chromatin condensation, nuclear fragmentation, and the formation of apoptotic bodies are typical morphological changes that result from activated caspase-9 further cleaving and activating downstream caspase-3, which in turn cleaves a variety of cytoskeletal and nuclear proteins, including poly (ADP-ribose) polymerase (PARP) [71]. Bcl-2 family proteins function as a life–death balance switch in this process. Research reveals that in N2a cells, T-2 dramatically raises the ratio of pro-apoptotic protein Bax to anti-apoptotic protein Bcl-2, whereas DON can downregulate Bcl-2 expression in PC12 cells; upsetting this balance directly induces apoptosis [75,85]. A study on chickens found that in two of the five chicks exposed to OTA for 21 days, focal tubular cell growth, numerous adenoma-like formations, and Bcl-2-positive epithelial cells were observed in layers of the renal papilla and convoluted tubules [86]. In ZEN-exposed spermatogonia cells, there was a considerable rise in cleaved caspase-3, caspase-8, BAD, BAX, and phosphorylated p53 and ERK1/2, accompanied by a demonstrable release of cytochrome c from mitochondria [87].

Autophagy functions as a very context-dependent “double-edged sword” in mycotoxin toxicity, much like apoptosis. Through the process of autophagy, cells use lysosomes to break down their own damaged parts in order to preserve homeostasis [88]. Autophagy is frequently induced under mycotoxin stress, but it can play two roles: it can contribute to cell death or operate as a means of cellular self-defense. Its activation is frequently a defensive adaptive response. For example, increasing autophagy with the autophagy activator rapamycin lowers ROS levels and prevents apoptosis in N2a cells treated with T-2 [71]. On the other hand, utilizing the autophagy inhibitor chloroquine induces apoptosis in PC12 cells treated with DON. This suggests that mycotoxin-induced autophagy protects these models by removing damaged mitochondria and reducing oxidative stress. Its molecular indicators include increased expression of the autophagy initiator Beclin-1 and the transformation of the essential autophagy protein LC3 from type I to type II and its punctate aggregation within cells (representing autophagosome formation). Autophagy, however, may have the opposite effect when toxin damage is severe. Unchecked or excessive autophagy can damage cellular architecture and directly contribute to the execution of cell death. For instance, AFB1 therapy induces significant autophagosome production and mitochondrial damage in C. elegans and human intestinal cells [75,89]. Although the goal of mitophagy is to remove damaged mitochondria, this process may become dysregulated if the damage is too great. Research shows that in C. elegans, AFB1 upsets the redox balance regulated by the DAF-16 (FoxO transcription factor homolog)/SOD-3 pathway, which in turn triggers autophagy and compromises the integrity of the intestinal barrier [89]. This suggests that the execution of its ultimate negative impact is directly facilitated by abnormal autophagy activation.

Autophagy and apoptosis interact deeply and intricately, exchanging upstream signals and impacting one another via important nodes. A key link between the two is oxidative stress; ROS produced by mycotoxins can initiate both autophagy and apoptosis. Additionally, proteins such as Bcl-2 can bind to Bax to prevent apoptosis and to Beclin-1 to prevent autophagy. Downregulation of Bcl-2 may concurrently alleviate inhibition of both pathways in DON-treated PC12 cells, resulting in concurrent activation of autophagy and apoptosis [75]. Their forms of interaction are varied: autophagy mostly protects against the neurotoxicity of T-2 and DON by preventing apoptosis, but in other circumstances, excessive autophagy may work in concert with or even encourage apoptosis, ultimately resulting in cell death [90]. The outcome of this dynamic balance ultimately determines cell fate. The complexity of the mycotoxin toxicity network is highlighted by research that has shown the participation of different kinds of programmed cell death in addition to autophagy and apoptosis. For example, recent research has shown that DON can induce intestinal epithelial cells to undergo planned necrosis (necroptosis), a process controlled by RIPK1/RIPK3 and followed by a robust inflammatory response [91], providing a new dimension for understanding its intestinal toxicity (Table 3).

**Figure 1:**
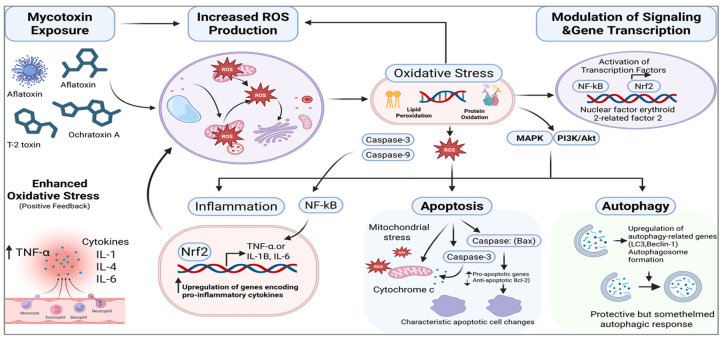
The mechanism of mycotoxin-induced oxidative stress, apoptosis, autophagy, and inflammation via activating ROS and modulation of gene transcription. Note: “↓” decrease; “↑” increase.

Autophagy and apoptosis provide a fundamental framework for understanding the molecular and cellular toxicity mechanisms of mycotoxins. Mycotoxins start these two intricately linked processes by destroying vital organelles like mitochondria. Future studies must examine important nodal molecules controlling their balance and thoroughly examine the fundamentals of this dynamic interaction in more accurate low-dose composite exposure models. For the purpose of thoroughly evaluating mycotoxin consequences and creating efficient preventative and control measures, it is essential to comprehend this cell death network at the systems level. The process of how mycotoxins induce oxidative stress, apoptosis, autophagy, and inflammation is shown in Figure 1.

### 3.3. Gut Microbiota Dysbiosis

The gut microbiota serves as an essential connection between these environmental contaminants and host metabolism [94]. Mycotoxins have a wide range of effects on the gut microbiome. Mycotoxin exposure can cause dysbiosis, which is characterized by microbial population changes and reduced diversity [95]. For instance, OTA has been shown to alter the gut microbiota in mice, even at low doses [96]. Similarly, DON and ZEN consumption can result in changes in the gut metaproteome of piglets, indicating functional changes in the microbial population [97]. These alterations could disturb the delicate balance of the gut environment, potentially resulting in a higher susceptibility to infections and reduced gut health.

The effect of AFB1 on the gut microbiota was assessed by removing the contents of broiler chickens’ duodenum after AFB1 exposure and using 16S rRNA sequencing to study changes in gut microbiota abundance and diversity. AFB1 at 1 ppm significantly (*p* < 0.05) reduced total LAB in broilers, according to a recent study [98]. The broiler group that received 1.5 and 2 ppm of AFB1 had a significant (*p* < 0.05) rise in Gram-negative bacteria and LAB counts. Furthermore, it was discovered that broilers exposed to 2.5 ppm of AFB1 produced greater total volatile fatty acids, showing a link between higher AFB1 doses and a higher incidence of LAB in the gut [99]. Giving pigs T-2 for one week was found to significantly increase the number of aerobic bacteria in their intestines [100]. Furthermore, T-2 at a dose of 1.6 mg/kg had negative effects on microbiota, which decreased beneficial *Bacillota* and *Muribaculum* and increased pathogenic *Pseudomonadota* and *Escherichia* [96]. T-2 has been shown to significantly impact bacterial populations; however, the precise mechanism underlying this transformation remains unknown. The modifications in the gut microbiota were evaluated utilizing the Biolog-Eco Plate method, which permits the quantification of culturable bacteria exclusively. Following six weeks of ZEA application, the results indicated a significant (*p* < 0.05) reduction in the levels of *E. coli*, *Enterobacteriaceae*, and *Clostridium perfringens* [101]. Capillary electrophoresis single-stranded conformation polymorphism (CE-SSCP) analysis reveals that fumonisins diminished the similarity of fecal microbiota SSCP profiles in pigs treated with fumonisins compared to the untreated control group. The results demonstrated an increase in microbial diversity [102]. Furthermore, at the genus level, OTA elevates the abundance of *Lactobacillus* while diminishing the populations of *Bacteroides*, *Dorea*, *Escherichia*, *Oribacterium*, *Ruminococcus*, and *Syntrophococcus*. The results indicated that lactobacillus exhibited greater resistance to OTA and may play a role in the detoxification of OTA. Furthermore, it was reported that OTA treatment positively affects facultative anaerobes [96]. This may indicate that the OTA could alter the gut microbiota in a way that is harmful to the host’s health.

Mycotoxin-induced gut microbiota dysbiosis decreases beneficial bacteria and increases pathogens. This imbalance weakens the intestinal barrier by breaking tight junctions and allowing for leakage. It also causes immunotoxicity through abnormal inflammatory responses and immune cell dysregulation, which eventually impairs systemic immunity and host defense. Future research should investigate microbiome-targeted therapies (probiotics, postbiotics) to restore barrier integrity and cure immunological dysfunction, progressing from observational correlations to mechanistic interventions.

### 3.4. Intestinal Barrier Damage Mechanism

The intestinal barrier is the first line of defense that keeps dangerous substances from entering the host’s body [14,103,104]. Maintaining general health depends on its structural integrity and regular operation [105]. However, a number of exogenous toxins have the potential to disrupt this delicate balance; mycotoxins, in particular, have attracted a lot of interest because of their high biological toxicity and ubiquitous prevalence. Mycotoxins can seriously affect the integrity of the intestinal barrier even at low concentrations, which can lead to systemic reactions, bacterial and endotoxin translocation, local inflammation, and poor nutritional absorption [17,106,107]. The intestinal barrier is a complex structure made up of microbial, chemical, immunological, and mechanical barriers. Among these, the primary physical barrier against luminal hazardous chemicals is the single layer of epithelial cells joined by tight junctions and the mucus layer that covers them [105,108]. Determining the cellular and molecular mechanisms by which mycotoxins break this barrier is crucial for evaluating the health consequences associated with them and for developing preventative measures.

One of the main mechanisms that many mycotoxins use to induce intestinal damage is oxidative stress. For instance, AFB1 induces severe oxidative stress in the intestine, which results in the translocation of the important antioxidant defense transcription factor DAF-16 (FoxO homolog) from the nucleus to the cytoplasm. This is followed by the suppression of expression of downstream genes, such as SOD-3, which reduces the antioxidant capacity of the cell [89]; knocking down the SOD-3 gene further aggravates AFB1-induced increased intestinal permeability and developmental toxicity, confirming the critical role of SOD-3-mediated antioxidant defense in barrier protection [89]. Intestinal barrier homeostasis is directly compromised by the “double hit” of a weakened antioxidant system and accumulating oxidative products, which result in extensive damage to cell membrane lipids, proteins, and DNA.

Mycotoxins directly target the junctional proteins connecting intestinal epithelial cells in addition to the extensive cellular damage caused by oxidative stress. A precise “gating” system that tightly controls paracellular permeability is formed by junctional proteins, which include occludin, members of the claudin family, and cytoplasmic plaque proteins like ZO-1 [109]. Research shows that intestinal epithelial cells exposed to toxins like AFB1 exhibit marked downregulation or altered distribution of important junctional proteins like Occludin and ZO-1 [89], thereby physically compromising the barrier’s seal. Additionally, some mycotoxins can target the cytoskeleton directly. According to research, they can disrupt microvilli-stabilizing proteins like ACT-5, which can induce microvilli to shed and impair intestinal epithelial absorption and barrier function, much like bacterial virulence factors [105]. The discovered cytoskeleton disruption process provides a possible viewpoint for comprehending the toxicity of particular mycotoxins (such as some Fusarium toxins), even though this study concentrated on bacteria. The relationship between mycotoxin exposure and tight junction disintegration is not a result of passive degradation but rather an active, signaling-mediated process. Deoxynivalenol (DON), the most common trichothecene and the most well researched mycotoxin in intestinal models, induces preferential activation of mitogen-activated protein kinase (MAPK) pathways, particularly ERK1/2, p38, and JNK, in jejunal IPEC-J2 cells [110]. This elucidates the dose- and time-dependent nature of barrier damage: at low concentrations, only redistribution transpires (reversible); at elevated concentrations, degradation prevails (less reversible). In vivo investigations in laying hens subjected to DON demonstrated reduced villi length, crypt hyperplasia, and compromised tissue integrity in the intestinal epithelium [111]. Morphological damage eliminates whole cells from the epithelial layer, resulting in holes that completely circumvent the paracellular route. FB1 functions via a unique mechanism. It obstructs ceramide synthase 2 (CerS2), hence altering sphingolipid metabolisms. Depletion of CerS2 induces endoplasmic reticulum (ER) stress, thereby activating apoptotic pathways and compromising the intestinal epithelial barrier [112]. This lipid-centric mechanism is exclusive to fumonisins and elucidates their specific toxicity to rapidly proliferating intestinal epithelial cells, which necessitate elevated sphingolipid turnover. In actual feed contamination, numerous mycotoxins coexist. Concomitant exposure frequently results in synergistic, rather than only additive, harm. Transcriptomic and proteomic analyses of Caco-2 cells subjected to simultaneous exposure to AFM1 and OTA demonstrated a synergistic effect on intestinal epithelial integrity that surpassed the impact of each toxin individually [113]. Likewise, the simultaneous exposure to cadmium and OTA resulted in increased barrier dysfunction in both Caco-2 and pig small intestinal epithelial (PSI) cells [114]. This synergy presumably occurs because various toxins simultaneously target distinct molecular sites—one may inhibit claudins, another may deplete mucin, and a third may induce inflammation, overwhelming the cell’s compensating mechanisms.

The presence of a single toxin alone is extremely uncommon in real-world natural contamination and dietary exposure settings; mixed exposure to many mycotoxins is common, and their combined toxic effects frequently considerably outweigh the simple sum of individual toxins. The primary proof is provided by a recent study conducted by the Chinese Academy of Agricultural Sciences, which shows that when AFB1 and its hydroxylated metabolite AFM1 act together on mice, their detrimental effects on intestinal barrier integrity and function are much stronger than the sum of the effects induced by individual exposure [115]. The harmful mechanisms of these two toxins overlap but have varied emphasis, according to a comprehensive proteome analysis: AFM1 predominantly impacts protein synthesis and transport, whereas AFB1 primarily interacts with pathways associated with lipid metabolism. When they coexist, they worsen each other’s metabolic disruptions and further limit the cell’s ability to heal itself, which results in more serious structural damage and functional loss of the intestinal barrier. This result cautions that risk assessment for food and feed safety and animal husbandry must place a high importance on the synergistic amplification effects of numerous coexisting mycotoxins. The influence of mycotoxins on the intestinal barrier is shown in Table 4.

Mycotoxins systematically damage the intestinal barrier by causing oxidative stress and directly breaking down tight junctions, among other molecular pathways. The synergistic toxic effects that result from the coexistence of numerous mycotoxins, which significantly increase the actual health threat, represent a more complex and realistic concern. Even if research on particular pathways and models has advanced, more work needs to be conducted to fully comprehend the signaling network as a whole, the fine regulation of junctional proteins, and the regulating impact of the gut microbiota. The intricate toxicity network will be further clarified by future studies using organoids, multi-omics, and composite exposure models. This will provide a strong scientific foundation for the development of precise detoxifying agents or barrier protectants, ultimately ensuring food and feed safety and public health.

### 3.5. Immunotoxicity Mechanism

Immunotoxicity is one of the most obvious adverse effects of mycotoxins which can result in immunosuppression, inflammatory imbalance, or even autoimmune reactions [118,119]. This process involves complicated interference with both the innate and adaptive immune systems, and its mechanisms can be carefully studied at the cellular and molecular levels. Mycotoxins first inflict significant damage to the innate immune system. Physical barriers and innate immune cells constitute innate immunity, which serves as the initial line of defense. For example, DON can disrupt intestinal epithelial tight junctions and increase intestinal permeability [120]. Deeper mechanistic studies reveal that particular protein toxins released by fungi can physically break cell membranes, sending powerful danger signals. Alternaria fungi produce Aeg-S and Aeg-L proteins, which can form transmembrane holes in respiratory epithelial cell membranes. In addition to directly causing cell damage, this membrane breach serves as the main trigger for abnormal immunological reactions [121]. Concurrently, important innate immune cells are directly inhibited by mycotoxins. By particularly upregulating the apoptotic pathway in neutrophils and targeting the Annexin A1 protein, OTA exposure dramatically lowers the numbers of neutrophils and macrophages, resulting in immune cell exhaustion [122]. AFB1 can compromise immune surveillance by impairing dendritic cell function and inhibiting natural killer cell activity [123]. Furthermore, by disrupting intracellular homeostasis pathways, mycotoxins frequently induce toxicity. ROS burst induced by AFB1 metabolism can impede antioxidant pathways such as SKN-1/Nrf2, hastening immunological senescence. Low-dose T-2 can encourage the translocation of gut opportunistic pathogens to the liver, activating the NOD2 pathway in Kupffer cells, causing local autophagy and a chemokine storm, and disrupting the liver immune microenvironment. Recent studies on T-2 have also demonstrated its mediation of remote immune disorders via the “gut–liver axis” [124].

At the adaptive immunity level, mycotoxins have complicated conflicting effects, often tending to inhibit protective immunity while abnormally stimulating pathogenic responses. One distinguishing aspect is the creation of a Th2-type immunological bias, which is closely associated to allergy disorders [21]. The Aeg-S/Aeg-L protein combination was found to “specifically” drive pulmonary eosinophil infiltration, Th2 cell accumulation, and raised IgE levels, while not encouraging normal Th1 or Th17 anti-infection responses [121]. Gene knockout tests demonstrated that these two genes are key factors of fungal-induced allergic airway inflammation. Mycotoxins, on the other hand, are more likely to have widespread immunosuppressive effects. AFB1 and OTA may limit T lymphocyte proliferation, diminish cytokine secretion, such as IL-2, and induce lymphocyte death, resulting in weaker vaccination responses and decreased resistance to reinfection [125]. According to the literature, exposure to composite mycotoxins can depress both innate and adaptive immune processes, increasing host susceptibility to infectious illnesses [126].

Common molecular hubs exist in mycotoxin immunotoxicity processes that span innate and adaptive immunity, increasing local harm signals and leading to systemic immune dysregulation. The main shared mechanism is the release and transmission of “danger signals.” Membrane rupture, necrosis, and oxidative stress all induce the release of endogenous alarmins such as IL-33. Research shows that after Aeg-S/Aeg-L pore formation the lung epithelium, IL-33 release is triggered via calcium influx and MAPK pathway activation, subsequently activating type 2 innate lymphoid cells (ILC2s) and Th2 cells, fully linking the pathological chain from physical damage to allergic response [121]. Second, dyregulation of apoptosis is a common hazardous outcome. OTA-induced neutrophil apoptosis reduces frontline defense, whereas excessive lymphocyte apoptosis leads to immunological organ atrophy and lower cell counts, perhaps raising autoimmune risk through greater release of self-antigens [122].

Mycotoxins form an immunotoxicity network that spans innate to adaptive immunity via a variety of mechanisms such as direct cytotoxicity, immune cell function suppression, danger signal transmission, and microbiome interference. Current research trends shift from single toxins to a systems perspective, concentrating on composite exposure and host–microbe interactions. Future research directions consist of using technologies such as organoids and multi-omics to unravel the network of toxin interactions and establishing targeted intervention techniques based on mechanisms to more effectively address the continued threat of mycotoxins to global health and food safety.

### 3.6. Endocrine Disruption, Epigenetic Changes, and Carcinogenic Mechanisms

The endocrine disrupting effects and carcinogenic dangers of long-term, low-dose mycotoxin exposure have emerged as major public health concerns. Their toxicity mechanisms comprise a complicated network including oxidative damage, genotoxicity, hormonal interference, and epigenetic changes. As previously mentioned, mycotoxin-induced cellular toxicity begins with oxidative stress and organelle destruction. Mycotoxin carcinogenesis is directly attributed to DNA damage and genotoxicity. Different toxins harm genetic material via various methods. Consider AFB1, one of the most potent natural carcinogens: after metabolic activation by cytochrome P450 enzymes, the resulting epoxide can covalently link to guanine bases in DNA, generating AFB1-N7-guanine adducts. The International Agency for Research on Cancer (IARC) classifies it as a Group I human carcinogen due to its direct genotoxicity, which can induce replication errors and DNA strand breaks [127]. In contrast, OTA, patulin (PAT), and others more typically induce DNA single-strand or double-strand breaks via indirect routes such oxidative stress [128], exhibiting characteristic “comet tail” abnormalities in comet assays. ZEN inhibits AFB1 toxicity in rat mammary glands, ovaries, livers, and spleens and its mechanisms [129].

Endocrine disruption effects are important ways by which certain mycotoxins promote the development of hormonally dependent malignancies. These toxins, due to their structural similarities to endogenous hormones, can disrupt endocrine system homeostasis. ZEN, a typical mycoestrogen, can bind to estrogen receptors (ERα/ERβ), imitating or antagonizing estradiol actions, encouraging aberrant proliferation of estrogen-sensitive cells in breast, ovarian, and other tissues, raising cancer risk [130]. Alternariol (AOH) has also been shown to exhibit estrogenic action. Furthermore, mycotoxins can disrupt hormone synthesis and metabolism. For example, AFB1 exposure significantly decreases estradiol levels in animal serum and mammary tissue, and when AFB1 and ZEN are co-exposed, they generate synergistic interference, further disturbing sex hormone balance and enhancing the carcinogenic risk.

Epigenetic changes offer a new way to understand mycotoxin toxicity over time and across generations. Epigenetic mechanisms affect gene expression without modifying the DNA sequence and are reversible [131,132]. Recent research showed that AFB1 dramatically lowers the overall N6-methyladenosine (m6A) methylation level of RNA in rat tissues. The mechanism involves downregulating methyltransferase expression, for example METTL3, and upregulating demethylase expression, for example FTO, which dynamically reconfigures the m6A modification landscape. m6A alteration is widely involved in mRNA splicing, stability, and translation; its perturbation may systematically affect the expression of genes associated with the cell cycle and apoptosis, suggesting a new mechanism for AFB1 long-term exposure carcinogenesis from the “epigenetic reprogramming” level [133]. Fumonisin B1 (FB1), a prevalent contaminant in maize-derived animal diets, causes worldwide DNA hypomethylation. The mechanistic pathway entails FB1’s established inhibition of ceramide synthase (sphingolipid biosynthesis), which impedes sphingolipid-dependent signaling; subsequently, this modifies the expression and activity of DNA methyltransferases (DNMTs), the enzymes tasked with preserving methylation marks [134]. The result is chromatin instability, leading to the derepression of transposable elements and the inappropriate activation of oncogenes that should stay inactive, thus contributing to neoplastic transformation. FB1 elevates the activating modification of H3K4me3 (trimethylation of histone H3 at lysine 4) at the PTEN promoter, hence augmenting PTEN transcription. This appears oddly protective: PTEN is a tumor suppressor that typically inhibits the pro-survival PI3K/AKT pathway. FB1 concurrently upregulates miR-30c, which inhibits PTEN translation at the mRNA level. The outcome is elevated PTEN mRNA levels coupled with diminished PTEN protein levels, resulting in persistent activation of PI3K/AKT signaling and compromised regulation of the DNA damage checkpoint [135]. This sophisticated yet harmful method demonstrates how mycotoxins orchestrate epigenetic alterations at many stages (histone → transcription, miRNA → translation) to produce a singular carcinogenic result. Patulin, generated by Penicillium species and a problem in fruit-derived animal diets, upregulates both DNMT1 (maintenance methyltransferase) and MBD2 (demethylase), resulting in a paradoxical condition where certain loci acquire and others diminish methylation. The outcome is impaired α-1 and α-2 adrenergic receptor signaling pathways, leading to renal damage [135]. ZEN, a potent mycoestrogen frequently found in swine and cattle feeds, and DON both alter miRNA profiles in ways that affect reproductive and immune cell function [136,137]. Mycotoxin-induced epigenetic alterations have been associated with disruptions in germ cell maturation and embryonic development, suggesting the potential for transgenerational epigenetic inheritance, although this is still a subject of ongoing research in livestock.

Multi-omics integration studies have revealed a systemic network of mycotoxin carcinogenesis and immune microenvironment alteration. Moving beyond single molecular activities, a systems biology view exposes more intricate interaction networks. For example, multi-omics (transcriptomics, single-cell sequencing) research of AFB1 combined with Mendelian randomization studies discovered that it can directly induce abnormal expression of core cell cycle genes, for example MAPK3 and CCNE1, resulting in a loss of cell cycle control [138]. In addition, the study showed that AFB1 target genes are highly active in particular T cell subsets within the tumor microenvironment: cytotoxic T cells with high MAPK3 expression show a functionally exhausted state at the invasive margin, while regulatory T cells with high CCNE1 expression may form an immunosuppressive barrier in the tumor core [138]. This suggests that mycotoxins can alter the tumor immunological environment in addition to directly harming parenchymal cells, fostering the growth of cancer cells and immune evasion.

Mycotoxin-induced endocrine disruption and carcinogenic processes represent a gradual, interconnected process ranging from molecular damage to cellular failure. In order to provide a scientific foundation for risk assessment and accurate prevention and control, future research should focus more on the combined exposure effects of multiple mycotoxins, the health risks of long-term low-dose exposure, and the use multi-omics technologies to identify important biomarkers and intervention targets.

## 4. Targeted Nutritional Regulation Strategies Based on Toxicity Mechanisms

In livestock nutrition, mycotoxin inactivation products are frequently used as nutritional interventions. In recent years, nutritional intervention techniques have progressed from simple adsorbents to advanced mycotoxin-inactivating compounds with bio-transformation capacities. Poultry is very susceptible to mycotoxins, and the presence of several mycotoxins in feed is highly prevalent. Nutritional interventions are primarily intended to reduce the oxidative stress, intestinal barrier dysfunction, and immunosuppression caused by mycotoxins. Pets have longer lifespans than domestic animals and poultry and consume dry feed based on grain processing for an extended period of time, putting them at a higher risk of chronic mycotoxin exposure. Given pets’ special standing in households, their nutritional intervention programs place a greater emphasis on safety and long-term success.

A study on early-lactation dairy cows found that a diet with ZEN (366.63 μg/kg dry matter), DON (1141.54 μg/kg dry matter), and FBs (613.49 μg/kg dry matter) significantly suppressed postpartum ovarian function. The first dominant-follicle ovulation rate was only 18%, compared to 78% in the control group. However, supplementation with a mycotoxin inactivation product slightly alleviated these adverse consequences, raising the ovulation rate to 50% and demonstrating improvements in indications such as the quantity of ovarian corpora lutea [139]. This study indicates that even when mycotoxin concentrations are below typical alarm thresholds, they can alter the endocrine axis and affect reproductive performance in livestock. A study on broilers found that providing contaminated feed substantially decreased weight gain and feed conversion efficiency; however, dietary supplementation with silymarin effectively mitigated these negative effects. Specifically, the silymarin-supplemented group not only improved growth performance but also reduced blood ALT activity, a biomarker of liver damage, and reduced mycotoxin-induced meat quality deterioration (e.g., greater cooking loss). Furthermore, silymarin treatment increased the meat’s polyunsaturated fatty acid content [140]. Notably, since dogs are very vulnerable to certain toxins (such as AFB), nutritional intervention should be part of an integrated prevention and control plan, along with raw material screening and regular monitoring, rather than the single approach [35]. Targeted dietary supplements are useful countermeasures. These products often contain specialized microbes or specific substrates with enzymatic activity, capable of converting poisons into less hazardous metabolites, therefore protecting animal reproductive systems and overall health.

### 4.1. Targeted Regulation Against Oxidative Stress and Novel Cell Death Mechanisms

Recent research has identified a variety of focused regulatory techniques. Among them, “ferroptosis” has received attention as an iron-dependent type of controlled cell death characterized by the accumulation of lipid peroxides. For the first time, researchers have proven that mycotoxins can induce “ferroptosis” in liver and intestine cells by altering intracellular iron metabolism and accelerating ROS bursts and lipid peroxidation. This work clarified the new process and identified nuclear receptor protein RORγ as the key regulatory target. Based on this target, the scientists screened isoorientin from the natural compound library using molecular docking technology. Experiments show that isoorientin activates RORγ, boosts antioxidant defense, blocks lipid peroxidation, and inhibits “ferroptosis” (ferroptosis) [141].

In addition to ferroptosis, “necroptosis” is a key programmed necrosis process. Concerning the intestinal damage generated by DON, it was discovered that DON can abnormally downregulate the expression of histone H3K9 methyltransferase SETDB1, and the absence of SETDB1 directly leads to necroptosis of intestinal epithelial cells [91]. This identifies a novel epigenetic target for intervention. The study revealed that pretreatment with certain pharmacologic necroptosis inhibitors (such as Nec-1) can greatly reduce cell death, intestinal tight junction disruption, and inflammatory reactions produced by DON [91]. This shows that finding natural active compounds that can upregulate SETDB1 expression or imitate its activity is an important path for future targeted nutritional management. Furthermore, a study found that when hens were simultaneously dosed with AFB1 and an extract of Sea Buckthorn (SB) berries, subsequent histology of the liver showed a significant reduction in necrosis and fatty accumulation compared to chickens treated with AFB1 alone. Immunohistochemical results showed that COX2, Bcl-2, and p53 were strongly expressed in the livers of AFB1-treated chickens, and SB oil supplementation dramatically reduced their expression. SB oil significantly reduced AFB1 residue levels in chicken livers, from 460.92 ± 6.2 ng/mL in the AFB1 group to 15.59 ± 6.1 ng/mL in the AFB1 and SB oil groups. These findings demonstrate that SB oil has a potent hepatoprotective function, lowering the concentration of aflatoxins in the liver and minimizing their harmful consequences [142].

In practical applications, adding antioxidants to feed is a popular strategy for reducing mycotoxin toxicity. Vitamins A, C, E, selenium, methionine, carotenoids, chlorogenic acid, and other antioxidants have all been shown to have protective properties [143,144]. Antioxidants such as selenium, vitamin E, and vitamin C can protect the spleen and brain from T-2 and DON-induced cell membrane damage. Chlorogenic acid and its derivatives have better free radical scavenging capacities than vitamin E and vitamin C, effectively eliminating DPPH radicals, hydroxyl radicals, and superoxide anion radicals, and can decrease the mutagenicity of AFB1 by reducing enzyme activation. Furthermore, using microorganisms such as lactic acid bacteria and acetic acid bacteria to degrade toxins or supplementing high-quality proteins and vitamins (A, D, E, K) in feed are both effective ways to prevent mycotoxin poisoning [145]. The antioxidant properties of various natural active components have also been demonstrated in animal models. Studies have shown that exogenous addition of Eucommia ulmoide flavonoids (EUFs), resveratrol, epigallocatechin gallate, and other antioxidant components can reduce DON-induced intestinal oxidative stress damage by restoring the body’s redox balance. For instance, DON can dramatically lower the activities of GSH-Px and SOD in piglets’ serum, whereas EUFs can dramatically lower the number of ROS and MDA in the serum. This suggests that EUFs can counteract the ROS imbalance brought on by DON by increasing the activity of blood antioxidant enzymes, which lowers oxidative stress in the body [146].

Additionally, lycopene (LYC) has been shown to be beneficial in reducing oxidative damage induced by mycotoxins. ZEN has been shown to generate intestinal oxidative stress in pregnant sows’ intestines by decreasing SOD, GSH-Px activity [147,148], and total antioxidant capacity (T-AOC) while increasing MDA levels. Previous research has shown that pretreatment with LYC may induce SOD and GSH-Px activity, as well as T-AOC content, in the small intestine of stressed male mice while decreasing MDA content. Similarly, adding 400 mg/kg LYC to pregnant sows’ diets can increase antioxidant indices in the small intestine and lower MDA levels, reducing oxidative damage induced by ZEN. Turmeric powder mitigated oxidative stress and decreased aflatoxin B1 (AFB1) levels in the liver of broiler hens subjected to EU-maximum contamination levels of AFB1 [149]. In aquaculture species, curcumin mitigated ochratoxin A (OTA)-induced muscular toxicity in grass carp (Ctenopharyngodon idella) via both in vitro and in vivo routes [150]. A different mycotoxin deactivator blend containing silymarin improved performance metrics by enhancing body weight, average daily gain, and feed intake, while also diminishing oxidative stress in 24-day-old weaned pigs exposed to 4.5 mg/kg of deoxynivalenol, 0.5 mg/kg of zearalenone, and 18 mg/kg of fusarium toxins [151].

### 4.2. Target Regulation for Gut Microbiota

A balanced gut microbiota is crucial for digestion, immunological function, and overall health in animals. Methods including probiotics, prebiotics, and postbiotics (PPSPs) are being investigated to restore gut microbial equilibrium and improve the detoxifying functions of the microbiota [152].

Probiotics are live bacteria that provide health benefits to the host when consumed in sufficient quantities. In the realm of mycotoxin mitigation, probiotics, especially certain strains of *Lactobacillus* and *Bifidobacterium*, are increasingly acknowledged for their capacity to bind mycotoxins, improve their biotransformation, and strengthen the gut barrier [153,154]. Probiotics in livestock have proven effective in mitigating the adverse effects of mycotoxins. Specific lactic acid bacteria and yeasts can biodetoxify mycotoxins in contaminated feedstuffs, providing a safe and effective method for mycotoxin reduction [155,156]. For pigs exposed to DON, probiotics have been shown to protect gut microbiota and overall health [157]. They can regulate the gut microbiome, reduce inflammation, and strengthen the intestinal epithelial barrier. In pets, including dogs and cats, gut health is fundamentally associated with general well-being, and probiotics are investigated as a method to alter the gut microbiota and enhance gastrointestinal health [158]. Although research on companion animals is developing, the principles of competitive exclusion, resource competition, and chemical communication by probiotics are universally applicable across species in influencing the gut microbiome.

In livestock, prebiotics enhance intestinal health, promote growth, bolster immunity, and modulate microbiota. Prebiotics can indirectly facilitate mycotoxin biotransformation and reduce their absorption by promoting the proliferation of good bacteria and enhancing the gut barrier [154]. The administration of lactulose, a prebiotic, has demonstrated ecological effects on the intestinal microbiota of piglets consuming a diet polluted with *Fusarium* toxins [159]. The fermentation of prebiotics by intestinal bacteria generates short-chain fatty acids (SCFAs) such as butyrate, acetate, and propionate. Butyrate is essential for colonocytes, acting as their principal energy source, fortifying the intestinal barrier, and demonstrating anti-inflammatory properties [160]. Synbiotics integrate probiotics and prebiotics to provide a synergistic effect that amplifies their positive influence on gut flora and host health. Postbiotics, comprising non-viable microbial cells or their constituents (such as metabolites and cell wall fractions), present a promising strategy for the reduction in mycotoxins [161]. They can influence outcomes through direct contact with mycotoxins or by influencing the host’s immune system and gastrointestinal environment. A multicomponent mycotoxin detoxifying agent comprising modified zeolite, Bacillus subtilis, B. licheniformis, Saccharomyces cerevisiae cell walls, and silymarin has demonstrated efficacy in mitigating mycotoxicosis caused by AFB1 and OTA in broiler chicks [162]. Therefore, nutritional interventions can restore the gut microbiota, which is crucial for nutrient digestion, absorption, and immune responses to alleviate mycotoxicosis.

The gut microbiome substantially affects the destiny and toxicity of mycotoxins. Gut microorganisms can metabolize mycotoxins via several biotransformation pathways, transforming them into less poisonous, more hazardous, or differentially active compounds [95,163]. Certain human gut bacteria may convert and metabolize significant food-derived mycotoxins and disguised mycotoxins, such as mycotoxin-glucosides, thus releasing unconjugated mycotoxins with modified toxicity profiles [164]. This biotransformation may encompass activities such as de-epoxidation, hydrolysis, and acetylation, which are essential for alleviating the detrimental effects of these toxins [165]. The efficacy of these microbial detoxification systems can significantly vary among individuals owing to variations in gut microbial composition and activity. Certain anaerobic rumen and intestinal bacteria eliminate the 12,13-epoxy ring from trichothecenes (DON), transforming them into far less hazardous de-epoxy metabolites. The fundamental reason ruminants exhibit greater resistance to trichothecenes compared to monogastric animals is that the rumen bacteria facilitate this conversion prior to the toxin reaching the absorptive epithelium [166,167]. Lactonases can cleave the lactone ring of ZEA, hence abolishing its estrogenic action. Bacterial species capable of degrading ZEA have been isolated from rumen fluid and intestinal contents; however, not all transformation products are non-toxic, as some intermediates maintain some biological activity [168]. For AFB_1_, certain *Flavobacterium* and *Bacillus* species can cleave the difuran ring or modify the lactone moiety, reducing genotoxicity [169]. OTA is digested by microbial carboxypeptidases and lipases into the significantly less dangerous ochratoxin α, a process that occurs effectively in the rumen and large intestine of ruminants [170].

Due to the substantial influence of this bidirectional connection, research is increasingly concentrating on techniques to alleviate mycotoxin toxicity through modulation of the gut microbiota. These interventions aim to introduce beneficial microorganisms or substances that promote their growth, thereby strengthening the gut’s defenses against mycotoxins.

Understanding the “microbiota–mycotoxin axis” is essential for formulating effective strategies to safeguard human and animal health against the widespread risk of mycotoxin contamination. This encompasses not only direct detoxification methods but also tactics that preserve a robust and resilient gut microbiome, enabling it to withstand the detrimental impacts of mycotoxin exposure. Additional research is required to thoroughly clarify the individual microbial species and metabolic pathways implicated in mycotoxin biotransformation and to enhance microbiota-targeted therapies.

### 4.3. Targeted Regulation of Intestinal Barrier Damage and Stem Cell Dysfunction

One of the current research priorities is to repair tight junctions and improve barrier function. In piglet experiments, DON primarily attacks the intestinal tract, activating nuclear factor-κB (NF-κB) and triggering excessive release of intracellular reactive oxygen species and various immune-related inflammatory factors, leading to intestinal mucosal congestion, edema, villus atrophy, crypt cell necrosis or apoptosis, and inhibition of tight junction protein synthesis, thereby increasing intestinal permeability and ultimately [171]. Currently, exogenous addition of yeast cell walls, oligosaccharides, probiotics, functional amino acids, and Chinese herbal extracts might partially treat DON-induced growth suppression and physiological problems in pigs. Eucommia ulmoides, for example, rely heavily on flavonoids (EUFs) as an antioxidant. EUFs improve immunological function and antioxidant capacity by regulating cytokine release, including IL-2 and TNF-α [171]. In the paraquat-induced oxidative stress model of piglets, EUFs dramatically enhanced piglet growth performance and reduced intestinal oxidative stress damage and inflammatory responses.

The level of inflammatory factor secretion is an essential physiological measure of the severity of inflammatory damage in the body. DON may activate NF-κB, induce inflammation, and selectively increase mRNA expression of immune-related inflammatory factors [70]. Multiple studies have confirmed the link between the NF-κB signaling system and oxidative stress, which is important for the body’s antioxidant and anti-inflammatory functions. Exogenous addition of EUFs, arginine, baicalin, berberine hydrochloride, and other substances can alleviate the increase in inflammatory factor secretion induced by DON, significantly reducing the protein and gene expression levels of interleukin-8 (IL-8), IL-1β, IL-6, and IFN-γ in the small intestine and serum, thereby improving the animal’s intestinal tract or body [172,173].

Studies have created AFF1 knockdown monoclonal IPEC-J2 cell lines, C57BL/6 animal models, and DON challenge experiments in order to elucidate the regulation mechanism of AFF1 in DON-induced intestinal injury. Flow cytometry, TMRE staining, and ATP content detection showed that AFF1 gene deletion could improve mitochondrial function, increase ATP content, and raise mitochondrial membrane potential (ΔΨm) in comparison to the DON treatment group, thereby reducing the buildup of ROS brought on by DON. AFF1 deletion significantly reduced DON-induced cell apoptosis, according to data on apoptosis rate and apoptosis-related protein detection. In the in vivo model, AFF1 knockdown considerably reduced the weight loss brought on by DON exposure in mice, boosted the mice’s resistance to DON, and greatly improved the intestinal villus structural disorder brought on by DON. SOD, CAT, GSH-PX, and total antioxidant capacity (T-AOC) activities all returned to normal levels following AFF1 knockout from the aberrant activation state induced by DON. Lactate dehydrogenase (LDH) and MDA levels also returned to normal ranges, suggesting that AFF1 knockout can restore the redox balance in intestinal tissues. AFF1 gene deletion decreased DON-induced intestinal cell apoptosis, as further demonstrated by TUNEL labeling and apoptosis-related protein identification [76]. Mycotoxin-induced intestinal damage has also been shown to be mitigated by other plant active substances. According to earlier research, LYC can lessen stress-induced intestinal damage by boosting the activity of local antioxidant enzymes in the colon [174]. LYC mainly reduces the oxidative stress induced by ZEN in the jejunum of mice by raising the content of T-AOC and lowering the levels of ROS and MDA in the jejunum [70]. Similarly, LYC can lessen ulcerative colitis brought on by OTA and lower the colon’s mRNA expression of the pro-inflammatory factor IL-6. According to the study’s findings, adding 1 mg/kg ZEN to pregnant sows’ intestines can raise the levels of pro-inflammatory cytokines TNF-α and IL-1β while lowering the levels of anti-inflammatory cytokines IL-4 and IL-10. This suggests that ZEN can induce intestinal inflammatory reactions in sows. Because of its anti-inflammatory and immunomodulatory properties, resveratrol has also been shown to reduce the intestinal inflammatory response that ZEN induces in mice [70]. By improving the microbiota’s abundance and SCFA content, adding 400 mg/kg LYC to the diet can reduce the negative effects of ZEN on the intestinal damage of pregnant sows during the embryo implantation period, as well as the decline in reproductive performance, intestinal oxidative stress, and immune function damage induced by ZEN through its anti-inflammatory and antioxidant functions [174]. In many investigations with one-day-old ducks, doses of 400 and 500 mg/kg of curcumin showed protective effects on the liver and gut against acute damage caused by 0.06 and 0.1 mg/kg of AFB1. Curcumin inhibited the production of CYP450 and AFB1-DNA adducts in the liver, while also modulating gut microbiota, intestinal NLRP3 inflammasome, and the TLR4/NF-κB signaling pathways in the intestines of ducks [175,176]. Moreover, the combination of curcumin, silymarin, and clays has enhanced reproductive and performance metrics in sows and piglets subjected to various mycotoxins. In 366-day-old sows, reproductive metrics and litter traits were enhanced due to the administration of a multi-component mycotoxin-detoxifying agent. Additionally, in 28-day-old weaned piglets, body weight and average daily gain were improved, while mortality and hepatic and intestinal damage were significantly diminished [177,178]. In an in vitro investigation utilizing a bovine fetal hepatocyte-derived cell line (BFH12), curcumin alleviated the hepatotoxic effects of AFB1 by enhancing antioxidant activity and the anti-inflammatory response. Curcumin administration elevated antioxidant levels, MDA concentration, and NQ01 enzyme activity, while diminishing CYP3A activity, which is involved in the bioactivation of AFB1 [179]. In nursery pigs contaminated with 500 ppb of AFB1, a blend of silymarin and other phytogenics protected the liver and intestines from mycotoxin-induced damage, improving liver biomarkers and gut morphology [180]. Furthermore, in 25-day-old post-weaned pigs subjected to 0.35 mg/kg of ZEN and 0.5 mg/kg of T-2, silymarin supplementation diminished mycotoxin residues, augmented biochemical markers, and increased growth performance [181].

### 4.4. Targeted Regulation for Immune Toxicity and Multi-System Interference

Modern nutritional regulation tactics have moved from conventional broad-spectrum adsorption to tailored intervention focused on the core hub of the “gut–immune–distal organ axis” in response to the immunological toxicity of mycotoxins and the multi-system interference they induce. Mycotoxins’ disturbance of the gut microecology and the subsequent systemic inflammatory cascade are the main induces of their multi-system toxicity.

Modern nutritional management, based on the response mechanism, focuses on precisely preventing harmful pathways, mending the intestinal barrier, and re-establishing microbial homeostasis: First, the liver is protected by supplementing with prebiotics for microbial intervention, such as xylooligosaccharides, which specifically encourage the growth of beneficial bacteria and prevent the excessive growth of pathogenic bacteria brought on by toxins [124]. Second, the intestinal epithelium is nourished, barrier function is strengthened, and anti-inflammatory benefits are achieved by using probiotics and their metabolic products, such as boosting the production of short-chain fatty acids like butyrate [182]. Third, by using natural active components that target toxic pathways, it has been discovered that mycotoxins can damage organs by interfering with cellular iron metabolism (ferroptosis), and that the alkaloid isoliquiritigenin, which is extracted from lotus seed hearts, can precisely activate the core regulatory protein RORγ, thereby improving cellular defense and preventing the ferroptosis process [141]. Another study used 444 mg/kg of curcuminoids from turmeric or 300 mg/kg of curcumin with male 180-day-old broilers, demonstrating liver-protective effects and the ability to alleviate immune organ damage caused by the exposure to 1 mg/kg of AFB1 [183]. Furthermore, the administration of 0.2% curcumin functioned as an immunomodulatory and protective agent against the detrimental effects of AFB1 (2 ppm) on immune organs in male 151-day-old broiler chicks, including the thymus, spleen, and bursa of Fabricius, while enhancing performance metrics such as body weight, feed intake, and feed conversion ratio [184]. In one-day-old ducks, OTA induced heightened expression of apoptosis-related genes and activated the TLR4/NF-κβ signaling pathway, while simultaneously downregulating mitochondrial transcription factors A, B1, and B in the gut. Nonetheless, the detrimental effects caused by 2 mg/kg of OTA were mitigated by 400 mg/kg of curcumin, safeguarding intestinal integrity and function, modulating gut microbiota, and enhancing performance metrics [185,186].

A scientific and practical nutritional approach is offered for methodically reducing the immune toxicity and multi-organ damage induced by mycotoxins by incorporating targeted components like prebiotics, microbial metabolic products, and isoliquiritigenin and approaching from both the regulation of intestinal microecology and the intervention of cellular pathways. Furthermore, Table 5 shows certain nutrient strategies based on their mechanisms of action, experimental models, and levels of evidence during mycotoxin toxicity.

### 4.5. Mycotoxin Adsorption and Biodegradation

The most prevalent detoxifying strategy for mycotoxin contamination in feed production is physical adsorption [204,205,206]. Physical detoxification techniques include heat treatment, grain processing and cleaning, adsorbent adsorption, and irradiation. Although heat treatment can lower toxin levels, the high temperature used often has an impact on the nutritional value and processing quality of grains. In contrast, adding adsorbents to feed is a more typical approach. Mycotoxin adsorption involves the binding of mycotoxins to solid-phase agents, thereby preventing their absorption in the gastrointestinal tract and reducing their bioavailability [156,207]. The idea is to use materials to adsorb mycotoxins in the animal’s intestinal tract, minimizing toxin absorption and damage to the body. Activated carbon, montmorillonite, zeolite, and yeast cell walls are among the most common adsorbent materials. However, due to the varying physical properties of the materials, the adsorption effects of different adsorbents on distinct mycotoxins differ significantly. For instance, research employing an in vitro model of the pig digestive system discovered that activated carbon/zeolite had no discernible adsorption effect on DON but had adsorption rates of 88% and 44%, respectively, for AFB1 and ZEN. The majority of investigated bentonites and zeolites had good adsorption effects on AFB1 (apart from activated carbon products), according to certain in vitro investigations, while the adsorption rates for DON and ZEN were often quite poor, averaging only approximately 8%. Furthermore, some adsorbents, like some hydrated zeolites, have low selectivity and can adsorb micronutrients like vitamin C and vitamin E in addition to toxins. Thus, a key area of future research is creating novel adsorbents that effectively adsorb weakly polar toxins (such ZEN and DON) without compromising feed nutrients.

Biological detoxification techniques, which use microbes or the enzymes they release to change the chemical structure of mycotoxins, eliminate or convert their poisonous groups and therefore achieve detoxification, demonstrating better application possibilities than physical procedures. For instance, detoxification can be achieved via aflatoxin oxidase isolated from *Armillaria mellea* acting on the double bond of the furan ring of AFB1 and hydrolyzing the resultant epoxide product to generate the less toxic AFB1-8,9-dihydrodiol [169]. The growth performance decline and liver damage induced by the toxin were mitigated when this enzyme was added to the feed of broilers contaminated with AFB1. Similar results were seen when aflatoxin-degrading enzymes were added to the feed of beef cattle, and the reduction in toxin residue in tissues improved the quality of the meat. Furthermore, great progress has been achieved in the biological destruction of mycotoxins utilizing microorganisms: lactic acid bacteria may significantly suppress the synthesis of toxins [208], while Bacillus subtilis B1 has an 82% inhibition rate against *Aspergillus flavus* development. AFB1 can be broken down by up to 82% using a composite biological degradation agent composed of lactic acid bacteria, *Bacillus subtilis*, and yeast in a 2:1:2 ratio. This effectively reduces the negative effects of contaminated feed on broiler production performance and prevents the expression of genes linked to liver lesions. According to a different study, laccase isolated from white-rot fungi could degrade AFB1 at a maximum rate of 90.33%. For the biological regulation of mycotoxins, these studies offer a variety of useful instruments and distinct development paths.

## 5. Conclusions and Further Directions

In conclusion, mycotoxin contamination poses significant risks to food and feed safety and health since it induces oxidative stress, apoptosis, autophagy disruption, inflammation, and dysbiosis. These impacts induce cell and tissue damage, which contributes to disease progression. Nutritional strategies, particularly diets high in antioxidants and anti-inflammatory substances, probiotics, and prebiotics show promise in alleviating these negative consequences by lowering oxidative damage and controlling cell death and inflammation. Global food and feed security is seriously threatened by mycotoxin contamination, which is now facing a number of issues such as a broad range of contamination, flaws in conventional techniques, the appearance of new issues, and difficulty in technology transfer. Conventional methods of detection and detoxification face challenges such as low sensitivity or detrimental impacts on grain quality. The emergence of novel and intricate poisons presents increasingly intricate demands for the system of prevention and management [209].

Therefore, future solutions are shifting to an intelligent and green full-chain preventive and control system [209]. On the detecting end, rapid and accurate portable intelligent detection technologies are breaking through the bottleneck of on-site screening. In terms of prevention and control, research should focus on using functional microbial agents for biological control, inhibiting toxin-producing fungi in the soil, and combining green detoxification technologies such as enzyme degradation, nanomaterials, and cold plasma to efficiently and safely remove toxins. Furthermore, pollution warning models created using artificial intelligence and big data can achieve early danger prediction [210], and stronger global collaboration will aid in standardization and technology sharing. To summarize, incorporating comprehensive solutions such as field warning, green blocking, speedy detection, and efficient detoxification is the main way to handle this difficulty. Dietary techniques combined with mycotoxin risk management provide a holistic strategy to improving health resilience and reducing the harmful impact of mycotoxins

## Figures and Tables

**Table 1:** Mycotoxin prevalence based on the relative incidence in 5593 global maize grain samples evaluated between January 2018 and May 2024 for 54 mycotoxins by using ultra-high-performance liquid chromatography with tandem mass spectrometry (Alltech 37+ Analytical Laboratory, Nicholasville, KY, USA) [6].

Mycotoxin Types	Occurrence (%)
Various mycotoxins	
Fumonisin B1	11%
Fumonisin B2	11%
Fumonisin B3	8%
Deoxynivalenol (DON)	11%
3-AcDON	3%
15-AcDON	8%
DON-3-Glucoside	2%
T-2 Toxin	1%
HT-2 Toxin	1%
Aflatoxin B1	1%
Cyclopiazonic Acid	2%
Zearalenone	5%
Emerging mycotoxins	
Fusaric Acid	13%
Beauvericin	4%
Moniliformin	6%
Enniatin A/A1	4%
Enniatin B/B1	4%

**Table 2:** The biological relevance of mycotoxins that cause mucotoxicosis when consumed by animals and can affect overall health and production.

Mycotoxin Type	Animal Species	Impact
Aflatoxins	Poultry	Induce oxidative stress and severe liver damage in ducks [38]. Impair growth performance, suppress immunity, reduce vaccine efficacy, and compromise food safety for broilers [39]. Altered immune and metabolic gene responses in spleen in turkeys following AFB1 exposure [40].
	Swine	Cause inhibition of cell growth, immunosuppression, mutagenicity, oxidative stress, lipid peroxidation, and DNA damage, resulting in lesions in the liver, spleen, lymph nodes, kidneys, uterus, heart, and lungs [41].
	Ruminants	The Toll-like receptor 2 pathway is activated in liver cells, indicating the participation of the innate immune system in cattle [42].
	Pets	Cause acute hepatotoxicity, coagulopathies, icterus, hepatic necrosis, and potentially death in dogs [43].
Zearalenones	Poultry	Chickens and other poultry show notable resistance to ZEN. This insensitivity is explained by multiple converging mechanisms [44]. Chickens that were given a ZEN-degrading enzyme demonstrated efficient gastrointestinal degradation even at food levels of 400 μg/kg. However, the baseline toxicity in untreated birds was already low [45].
	Swine	Show vulvar swelling (hyperestrogenism), uterine enlargement, and premature mammary growth even at modest dietary doses [46,47]. Reduced birth weight, impaired muscle development in piglets, and altered expression of growth-related genes [48]. At levels close to the EU recommendation of 100 ppb, it causes oxidative stress and inflammatory signaling in the colon of piglets that have been weaned via changing the NF-κB and Nrf2 pathways [49].
	Ruminants	Ruminants are moderately sensitive to ZEN. The rumen microbiota helps protect the body by breaking down ZEN before it reaches the places in the intestines where it can be absorbed. The rumen changes ZEN, but how much it changes depends on the diet, the conditions in the rumen, and the animal itself [50].
	Pets	The EFSA scientific opinion determined a lowest observable adverse effect level (LOAEL) for dogs, signifying that dogs are one of the species for whom adverse effects have been recorded at specific dose levels. The likelihood of negative health consequences from a ZEA-containing diet was assessed as minimal for dogs. Nevertheless, no reference points could be identified for felines, indicating that the data are inadequate to comprehensively delineate feline sensitivity [51].
Deoxynivalenol	Poultry	Adverse effect thresholds of 1.9 mg DON/kg feed for broiler chickens and 1.7 mg DON/kg feed for turkeys were established, resulting in diminished villus height and histopathological damage. This resulted in updated reference thresholds for detrimental health impacts of 0.6 mg/kg feed for both broiler chickens and turkeys [52].
	Swine	DON impairs intestinal barrier function, damages jejunal morphology, and triggers inflammatory cascades [53], anorexia and emesis [54]. DON-exposed pigs show altered gut microbial composition that correlates with the degree of feed refusal [55].
	Ruminants	Although cattle are typically regarded as reasonably tolerant to DON due to detoxification by rumen microbes (conversion to DOM-1), intriguing cellular-level findings present a contrasting narrative. Bovine peripheral blood mononuclear cells exhibit sensitivity to DON in vitro, indicating that once DON evades rumen detoxification and enters systemic circulation, bovine immune cells become significantly susceptible [56].
	Pets	Information on DON toxicity specifically in dogs and cats is more limited than for livestock species.
Ochratoxins	Poultry	Enlarged, pallid kidneys exhibiting tubular degeneration, the most characteristic lesion; a reduction in both humoral and cell-mediated immunity, atrophy of the bursa of Fabricius, and heightened vulnerability to secondary infections [57]. Hepatomegaly, steatosis, and necrosis; a diminished development rate, lowered egg production, and compromised eggshell quality [58].
	Swine	Progressive injury to renal proximal tubules, regarded as the characteristic lesion; diminished lymphoproliferative responses and antibody levels; reduced feed consumption, decreased body weight increase, and a suboptimal feed conversion ratio [59].
	Ruminants	Cattle, sheep, and goats exhibit far greater resistance to OTA due to their rumen bacteria, which hydrolyzes OTA into the largely non-toxic compound OTα. This detoxification occurs swiftly and effectively with normal rumen function [59].
	Pets	Dogs and cats are considered “unusually susceptible” to the hepatotoxic, nephrotoxic, immunosuppressive, and carcinogenic effects of OTA.
Trichothecenes type A	Poultry	The T-2 toxin in poultry causes oral and intestinal lesions, impairs immune responses, damages the hematopoietic system, reduces egg production, thins egg shells, induces feed refusal, causes weight loss, alters feather patterns, results in abnormal wing positioning, and leads to hysteroid seizures or impaired righting reflex [60,61].
	Swine	The hallmark response is emesis followed by anorexia and reduced feed intake [62]. In conjunction with the serous–hemorrhagic necrotic–ulcerative inflammation of the digestive tract, necroses manifest on the snout, lips, and tongue, accompanied by edema and mucous coatings of the gastric mucosa, swelling in the head region, particularly around the eyelids and larynx, and infrequently, paresis or paralysis [63].
	Ruminants	T-2 toxin exposure has been associated with feed refusal, production losses, gastroenteritis lesions, intestinal hemorrhages and death in dairy cattle [63]. Experimental evidence indicates that lambs administered the T-2 toxin exhibit signs of localized hyperemia and dermatitis at the mucocutaneous junction of the lip commissure, accompanied by diarrhea, leukopenia, lymphopenia, and lymphoid depletion in the mesenteric lymph nodes and spleen [64].
	Pets	Dogs may encounter trichothecenes via contaminated ingredients in pet food. The literature concerning pet vulnerability to trichothecenes is markedly scarce. Delivery of the T-2 toxin in felines produced symptoms akin to those associated with alimentary toxic aleukia, a human condition resulting from the ingestion of grains contaminated with T-2. Clinical observations comprised emesis, hematochezia, dehydration, weight reduction, lethargy, ataxia, dyspnea, and anorexia. Additionally, bone marrow aplasia, variations in lymphatic tissue, bleeding diathesis, impaired hemostasis, and modifications in proliferative tissues were observed [65].

**Table 3:** The impacts of mycotoxins on apoptosis and autophagy.

Mycotoxins	Object of Study	Concentration	Toxic Effect	References
T-2 toxin	Mouse neuroblastoma cells N2a	5–80 ng/mL	Induction of ROS production Decreased mitochondrial membrane potential Activated the mitochondrial apoptosis pathway (with an increased Bax/Bcl-2 ratio and activation of caspase-9/-3)	[71]
T-2 toxin	Mouse microglial cells BV2	1.25–5 ng/mL	Dependence leads to ROS Mitochondrial dysfunction (Bax ↑,Bcl-2 ↓) Activation of the caspase-3/PARP-1 apoptotic signaling pathway	[92]
Deoxynivalenol DON	Rat pheochromocytoma cells PC12	125–2000 ng/mL	Inducing apoptosis through the mitochondrial pathway Bcl-2 ↓, Bax ↑, activation of caspase-3/-9 Activated the PIK3C3/beclin 1/Bcl-2 autophagy pathway	[75]
Aflatoxin B1 AFB1	Porcine Intestinal Epithelial Cells IPEC-J2	0, 5, 10 μg/mL	Induce ROS accumulation Structural damage to mitochondria and lysosomes Disrupted autophagic flux through the ROS/TRPML1 pathway	[93]
Deoxynivalenol DON	Porcine Intestinal Epithelial Cells IPEC-1	4 mg/kg	Induction of programmed necrosis	[91]

Note: “↓” decrease; “↑” increase; BAX (Bcl-2-associated X protein); ROS (Reactive oxygen species); TROML1 (transient receptor potential).

**Table 4:** The influence of mycotoxins on the intestinal barrier.

Mycotoxins	Object of Study	Concentration	Toxic Effect	References
Aflatoxin B1 + M1 AFB1 + AFM1	ICR mice Differentiated Caco-2 cells	In vivo: 0.3 mg/kg + 3.0 mg/kg In vitro: 4 μg/mL + 4 μg/mL	Reduces cell viability and transepithelial electrical resistance Downregulates the expression of tight junction proteins Induces their endocytic redistribution	[115]
Aflatoxin M1 + Ochratoxin A AFM1 + OTA	ICR mice Differentiated Caco-2 cells	In vivo: 0.12 μg/mL + 0.2 μg/mL 12 μg/mL + 20 μg/mL In vitro: 3.5 mg/kg + 3.5 mg/kg	Decreased expression of tight junction proteins Increased intestinal epithelial permeability Inflammatory cell proliferation and a reduction in goblet cells	[116]
Fumonisin B1 FB1	IPEC-J2 cells	80 μg/mL	Cell activity is inhibited Induce the release of lactate dehydrogenase Reduce the expression of tight junction proteins	[112]
Deoxynivalenol DON	Piglet	100 μg/kg	The expression level of intestinal tight protein connections decreases	[117]

**Table 5:** Dietary techniques reducing the negative effects of mycotoxins.

Feed Supplement	Dose	Model	Mode of Action	Levels of Evidence	References
Regulation against oxidative stress and novel cell death mechanisms					
Curcumin	300 mg/kg	One-day-old broilers	↓ ROS, and MDA. ↑ SOD, CAT, GSH, and ATPase	Reduced oxidative stress, and necroptosis, induced by AFB1 liver tissue at 1 mg/kg	[183]
	400 mg/kg	Broilers	↑ CAT, SOD2, and ABCG2	Decrease in hepatic AFB1 to undetectable levels (<LOD) at (0.02 mg/kg feed)	[149]
	222 mg/kg	Broiler chickens	↓ oxidative stress genes	Decrease in hepatic AFB1 in liver at (1.0 mg/kg feed)	[187]
Lycopene	25 mg/kg.bw/d	Male broiler	↓ ROS, MAPK and NF-κB signaling pathways	Inhibited oxidative stress and inflammation induced by T-2 toxin 1.5 mg/kg.bw/d	[188]
Compound mycotoxin detoxifier (CMD)	0.5, 1.0 and 1.5 g/kg CMD	One-day-old Ross broilers	↓ MDA, ↑ SOD, GSH-Px, CAT	Inhibited AFB1 toxicity at 40 μg/kg	[189]
Grape seed	250 and 500 mg/kg	One-day-old broiler	↓ oxidative stress	Reduced the oxidative stress caused by 400 mg/kg of FB1	[190]
	8%	Four-week-old weaned piglets	↑ SOD, CAT, and GSH	Reduced oxidative stress caused by 320 µg/kg of AFB1	[191]
	250 and 500 mg/kg	One-day-old Cobb chicks	↓ MDA, ↑ T-SOD, GSH-Px, CAT, GR, GST, and GSH	Reduced the oxidative stress caused by 1 mg/kg AFB_1_	[192]
Regulation for gut microbiota improvement					
Clove	0.1%, 0.2% and 0.5%	In vitro	↓ growth	Inhibit the growth of toxin producing *Aspergillus flavus* and *Aspergillus parasiticus*	[193]
Compound mycotoxin detoxifier (CMD)	0.5, 1.0 and 1.5 g/kg CMD	One-day-old Ross broilers	↑ *Staphylococcus-xylosu*, *Esherichia-coli-g-Escherichia-Shigella*, and ↓ *Lactobacillus-aviarius* abundance	Inhibited AFB1 toxicity at 40 μg/kg	[189]
Ginger	100 mg/kg/d 250 mg/kg/d	Male Wistar rats	↓ ROS, DNA strand break; ↑ Nrf2/HO-1	Inhibited AFB1 toxicity at 200 μg/kgA/2d	[194]
Plant-Derived *Lactobacillus plantarum* BCC 47723	10^9^ cfu/mL	In vitro	↓ ZEA from liquid medium	Inhibited ZEA toxicity at 0.2 µg/mL	[195]
*Bacillus velezensis* A2	2 mL	Mice	↑ SCFAs	Alleviate injury caused by ZEA (40 mg/kg BW) by regulating the intestinal flora and the content of short-chain fatty acids in the cecum	[196]
Regulation against the intestinal barrier damage and stem cell dysfunction					
Curcumin	500 mg/kg	One-day-old broilers	↓ Nrf2 signaling pathway	Protect the intestines from AFB1-induced damage (1 mg/kg)	[197]
	150 mg/kg, 450 mg/kg	One-day old broilers, 120-day-old broilers	↓ CYP450	Alleviated liver and intestinal damage caused by 100 µg/kg of AFB1 and 5 mg/kg of AFB1, respectively	[198,199]
Lycopene	200 and 400 mg/kg	One-day-old broilers	↓ IFN-γ, IL-1β, ↑ CLDN-1, and ZO-1	Alleviate 100 µg/kg of AFB1 toxicity in the gut and the liver	[200]
Compound mycotoxin detoxifier (CMD)	0.5, 1.0 and 1.5 g/kg CMD	One-day-old Ross broilers	↓ villi rupture	Inhibited AFB1 toxicity at 40 μg/kg	[189]
Regulation against the immune toxicity and multi-system interference					
Curcumin	300 mg/kg	One-day-old broilers	↓ TLR4/RIPK signaling pathway	Reduced inflammation induced by AFB1 in liver at 1 mg/kg	[183]
	0.2%	151-day-old broiler	↓ C-reactive protein (CRP)	Protective against the damaging effects of AFB1 (2 ppm) on immune organs such as the thymus, spleen, and bursa of Fabricius	[184]
	0.05%(*w*/*w*)/d	Male Fisher-344 rats	↑ GSHT, UGT1A1; ↓ CYP1A1, LDH, and ALT	Hepatoprotective effects against AFB1 toxicity at 20 µg/d	[201]
Epigallocatechin-3 gallate (EGCG)	5–20 μM EGCG	In Vitro	↓ NF-κB, COX-2, and caspase-3	Inhibited the toxic effects and inflammatory reactions induced by DON at 250–1000 ng/mL	[202]
Thyme oil	250/500 mg/d	Egyptian male sheep	↓ AST, ALP, and γGT	Mitigated the liver toxicity caused by AFB (10 mg/kg/d) and restore the overall performance of the sheep	[203]
Grape seed	250 and 500 mg/kg	One-day-old Cobb chicks	↑ IgA, IgG, and IgM	Enhanced immune response of birds exposed to1 mg/kg AFB_1_	[192]
	8%	Four-week-old weaned piglets	↓ IL-1 beta and TNF alpha	Reduced inflammation enhanced by 320 µg/kg of AFB1	[191]

Abbreviations: aspartate aminotransferase (AST); and alkaline phosphatase (ALP); gamma-glutamyl transferase (γGT); total antioxidant capacity (TAC); glutathione peroxidase (GPx); nuclear factor erythroid2-related factor2 (Nrf2); tumor necrosis factor-α (TNF-α); interleukin (IL-6); epigallocatechin 3-gallate (EGCG); cyclooxygenase-2 (COX-2); reduced (↓); induced (↑).

## Data Availability

No new data were created or analyzed in this study. Data sharing is not applicable to this article.

## Abbreviations

The following abbreviations are used in this manuscript:

AFB1	Aflatoxin B1
AFB1 + AFM1	Aflatoxin B1 plus aflatoxin M1
AOH	Alternariol
CAT	Catalase
DON	Deoxynivalenol
EUF	Eucommia ulmoides flavonoids
ERα/ERβ	Estrogen receptor alpha/beta
FTO	Fat mass and obesity-associated protein
GSH	Glutathione
GSH-Px	Glutathione peroxidase
HMGB1	High mobility group box 1
IARC	International Agency for Research on Cancer
ICR	used here as a mouse model strain
IPEC-J2	Intestinal porcine epithelial cell line J2
IL-2	Interleukin-2
IL-4	Interleukin-4
IL-6	Interleukin-6
IL-8	Interleukin-8
IL-10	Interleukin-10
IL-17	Interleukin-17
MDA	Malondialdehyde
MAPK	Mitogen-activated protein kinase
METTL3	Methyltransferase-like 3
NF-κB	Nuclear factor kappa-light-chain-enhancer of activated B cells
Nec-1	Necroptosis inhibitor
OTA	Ochratoxin A
PAT	Patulin
PARP	Poly (ADP-ribose) polymerase
RIPK1/RIPK3	Receptor-interacting protein kinases 1 and 3
RORγ	Nuclear receptor ROR gamma
SOD	Superoxide dismutase
SCFA	Short-chain fatty acid
SD	Sea Buckthorn
T-2:	Trichothecenes toxin
T-AOC	Total antioxidant capacity
TUNEL	Terminal deoxynucleotidyl transferase dUTP nick end labeling
ZEN	Zearalenone
